# Ultrasound-Mediated Mesenchymal Stem Cells Transfection as a Targeted Cancer Therapy Platform

**DOI:** 10.1038/srep42046

**Published:** 2017-02-07

**Authors:** Tom Haber, Limor Baruch, Marcelle Machluf

**Affiliations:** 1 Faculty of Biotechnology & Food Engineering, Technion – Israel Institute of Technology, Haifa, 32000, Israel

## Abstract

Mesenchymal stem cells (MSCs) hold tremendous potential as a targeted cell-based delivery platform for inflammatory and cancer therapy. Genetic manipulation of MSCs, however, is challenging, and therefore, most studies using MSCs as therapeutic cell carriers have utilized viral vectors to transduce the cells. Here, we demonstrate, for the first time, an alternative approach for the efficient transfection of MSCs; therapeutic ultrasound (TUS). Using TUS with low intensities and moderate frequencies, MSCs were transfected with a pDNA encoding for PEX, a protein that inhibits tumor angiogenesis, and studied as a cell vehicle for *in vivo* tumor therapy. TUS application did not alter the MSCs’ stemness or their homing capabilities, and the transfected MSCs transcribed biologically active PEX. Additionally, in a mouse model, 70% inhibition of prostate tumor growth was achieved following a single I.V. administration of MSCs that were TUS-transfected with pPEX. Further, the repeated I.V. administration of TUS-pPEX transfected-MSCs enhanced tumor inhibition up to 84%. Altogether, these results provide a proof of concept that TUS-transfected MSCs can be effectively used as a cell-based delivery approach for the prospective treatment of cancer.

Cell-based delivery systems are an exciting and promising therapeutic concept for the therapy of an array of disorders and malignancies, and are emerging as an alternative approach for viral gene-therapy and other targeted delivery systems^1^. Mesenchymal stem cells (MSCs), particularly bone marrow-derived MSCs, have been studied extensively for cancer cell-based therapy^2^,^3^,^4^ due to their natural homing ability to sites of injury and inflammation^2^,^3^,^5^. This homing ability allows the use of MSCs expressing exogenous anti-cancer proteins as drug delivery vehicles, which upon administration to tumor-bearing animals reach tumor sites and inhibit tumor growth^6^,^7^. Moreover, their hypo-immunogenicity^7^,^8^ and immunosuppressive properties^9^ may facilitate the clinical implementation of allogeneic MSC administration for a variety of clinical applications^7^. Most studies using MSCs as a therapeutic cell carrier have utilized viral-based vectors such as adenovirus, adeno-associated virus (AAV) or lentivirus to transduce the cells and achieve high continuous expression of the therapeutic agent when aiming to target tumors *in vivo*^10^. Nevertheless, as far as gene therapy in general, limitations associated with viral-based vectors such as safety, ease of preparation, immunogenic response and the size of the introduced gene must still be surmounted^11^,^12^.

In the current study, we assessed the feasibility of therapeutic ultrasound (TUS) as a potentially safe and efficient method to transfect MSCs with pDNA encoding for an inhibitor of tumor angiogenesis, and thus to produce a cell vehicle for *in vivo* tumor therapy. Ultrasound is a promising non-viral approach, which has been demonstrated to safely deliver genes into cells and nuclei^13^,^14^,^15^. Among the various ultrasound modalities used for gene delivery, therapeutic ultrasound (TUS, 1–3 MHz, intensities: 0.5–2 W/cm^2^, pulsed-mode) is considered safe in terms of cell and tissue damage and is approved for other clinical applications^16^. We previously reported the application of TUS to directly deliver pDNA encoding for hemopexin-like domain fragment (PEX) to tumors *in vivo*, thereby demonstrating the efficacy of TUS as a non-viral gene delivery technology^17^. PEX is a 29 kDa noncatalytic C-terminal fragment of MMP-2. PEX interacts with the endothelial cell’s αvβ3 integrin, and by this, serves as a natural inhibitor of MMP-2. It simultaneously inhibits angiogenesis, cell proliferation, and migration *in vitro* and *in vivo*^18^,^19^. We have also demonstrated that TUS is safe when used *in vivo* and can be used repeatedly to transfect tumors with pDNA^17^,^20^. The efficiency of TUS-transfection can be improved when using ultrasound contrast agents (USCAs; gas-filled microbubbles) such as Optison^TM^, which deliver the DNA to the cells and induce cavitation^21^,^22^. As we showed, USCAs enhance TUS gene transfection by increasing plasmid number in each cell but also by delivering plasmids to more cells. USCAs interacts with the DNA and mainly affect the cell cytoplasmatic membrane, without interfering with DNA intracellular trafficking^21^.

Our aim in the present study, therefore, was to transfect MSCs using TUS and pDNA encoding for PEX, and to utilize the transfected cells as a drug delivery vehicle, targeting most types of tumors. TUS technology has not yet been examined as a method for transfecting MSCs with pDNA, hence we also had to address its effect on the MSCs’ stemness and homing abilities. Most importantly, the effect of TUS-pPEX transfected-MSCs on tumor growth was studied as well as their repeated administration to mice bearing prostate tumors.

## Results

### MSCs express PEX following TUS-transfection with pPEX

**Figure 1:**
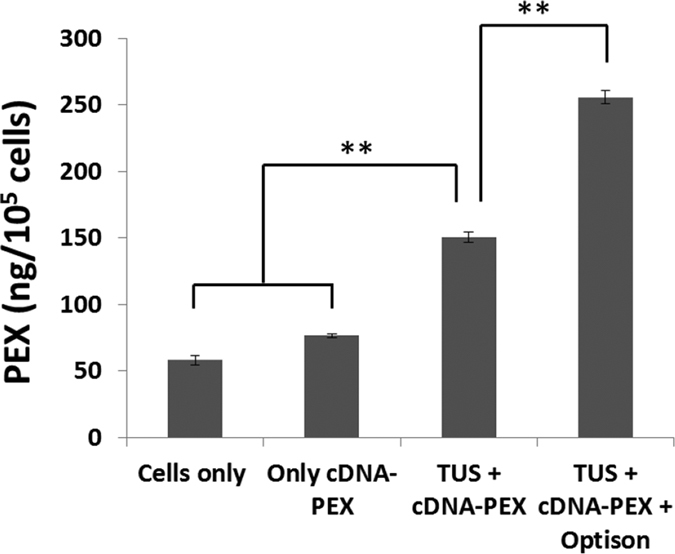
Effect of TUS transfection on PEX expression level in MSCs. PEX secreted to culture media of TUS-transfected MSCs with or without USCA (Optison) was quantified using ELISA and presented as ng PEX/10^5^ cells ± SD, **p < 0.001, n = 5.

To validate TUS-MSC transfection we first compared the transfection efficiencies obtained when using TUS and TUS + USCA to the ones obtained when using commercially available transfection reagents, and demonstrated that significantly higher transfection efficiency can be obtained using TUS + USCA while high levels of viability are preserved (Fig. S1). Following, the expression of PEX after TUS-MSC transfection with pDNA-PEX was assessed. Conditioned media harvested from TUS-transfected MSCs, non-transfected MSCs or MSCs incubated with pDNA-PEX without TUS application were assessed for the presence of PEX protein using ELISA. As seen in Fig. 1, the highest concentration of PEX was observed in MSCs, which were TUS + USCA-transfected with pDNA-PEX. The PEX level in these cells was 170% higher than TUS-MSCs transfected with pDNA-PEX but without USCA (p < 0.001). Nevertheless, the PEX expression level in TUS-transfected MSCs without USCA was significantly higher than the controls (p < 0.001).

### MSCs expressing PEX post TUS-transfection inhibit endothelial cells migration and proliferation and induce apoptosis

**Figure 2:**
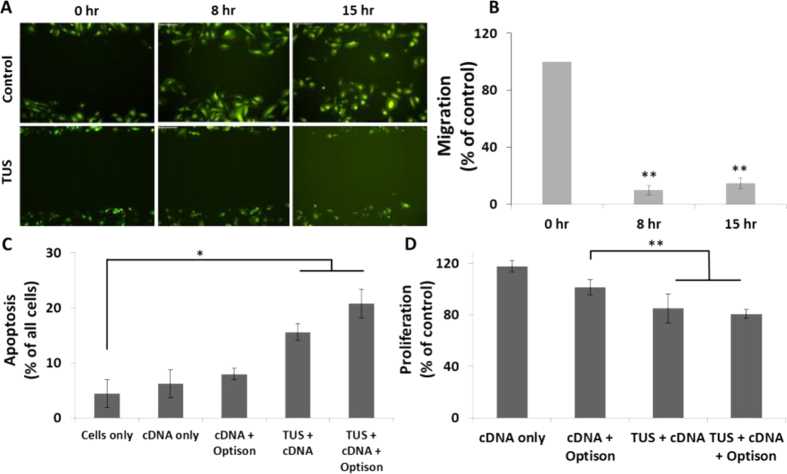
Effect of PEX from TUS-transfected MSCs with and without USCA (Optison) on the migration, proliferation and apoptosis of endothelial cells. (**A**,**B**) Migration assay of HUVECs after incubation in conditioned media of TUS-transfected MSCs at different time points. Percentage of migrated cells is compared to control. n = 9 (**C**) Apoptosis (n = 4) and (**D**) Proliferation (n = 6) of HUVECs after incubation with conditioned media of TUS-transfected MSCs, expressed as % of control ± SD. *p < 0.01, **p < 0.001.

The biological activity of PEX transcribed by MSCs post TUS-transfection was measured through its effect on HUVEC migration, proliferation, and apoptosis. Conditioned media taken from MSCs, three days after TUS + USCA-transfection with pDNA-PEX, led to a significant—85%— inhibition in endothelial cells *migration* after 15 hours (Fig. 2A,B). PEX transcribed from MSCs after TUS-transfection significantly induced *apoptosis* of HUVECs (Fig. 2C). Conditioned media of MSCs TUS-transfected with pDNA-PEX or TUS + USCA-transfected with pDNA-PEX resulted in significant increases in HUVEC apoptosis from 4% ± 3 (control) to 16% ± 2 and 21% ± 3, respectively (p < 0.01, Fig. 2C). These results contrast with the non-significant increases in apoptotic HUVECs that were incubated with conditioned media taken from MSCs treated with pDNA-PEX, or with pDNA-PEX and USCA without TUS application (6% ± 3 and 8% ± 1, respectively). HUVEC *proliferation* was also significantly inhibited when treated with conditioned media of MSCs TUS-transfected with pDNA-PEX with or without USCA (19% ± 3 and 15% ± 11, respectively, p < 0.001, Fig. 2D).

### MSCs secreting PEX affect cancer cells

**Figure 3:**
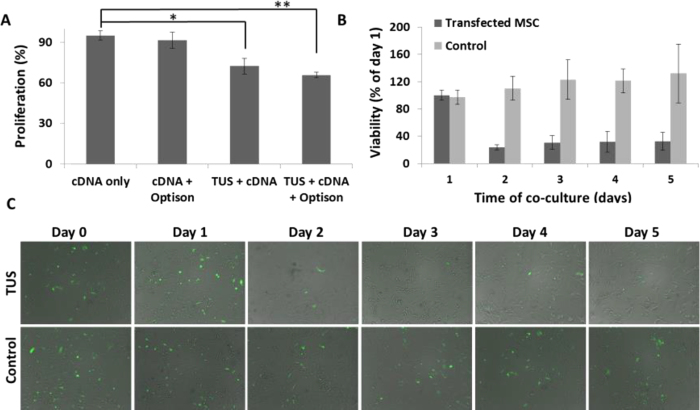
Effect of MSC-secreting PEX post TUS transfection with and without USCA (Optison) on the viability and proliferation of cancer cells. (**A**) Proliferation of PC3 cells after incubation with conditioned media of MSCs transfected using TUS (n = 7), (**B**) Viability of PC3 cells co-cultured with TUS-transfected MSCs along 5 days (n = 4). Results are presented as mean ± SD (**C**) Representative micrographs of PC3 cells (green) after being co-cultured with TUS-transfected MSCs. *p < 0.01, **p < 0.001.

PEX is known to inhibit the proliferation of cancer cells as well as endothelial cells^17^,^18^,^23^. In order to evaluate the effect of PEX on the proliferation of a human prostate cancer cell-line (PC3), the cells were incubated with conditioned media of MSCs TUS-transfected with pDNA-PEX with or without USCA. The proliferation of PC3 cells was inhibited by 28% using conditioned media of MSCs that were TUS-transfected with pPEX (p < 0.01, Fig. 3A). The addition of USCA resulted in an even higher inhibition of PC3 cell proliferation when compared to control (34% inhibition, p < 0.001). To further study the direct biological activity of the secreted PEX from TUS-transfected MSCs (using pDNA-PEX and USCA) on PC3 cells, MSCs were co-cultured with GFP-PC3 cells, 24 hr post transfection. As seen in Fig. 3B,C, a significant reduction of 76% in the number of viable GFP-PC3 cells was obtained after two days of co-culture, and the number of GFP-PC3 viable cells remained low for an additional three days. In the control co-cultured group, there was no significant change in the number of GFP-PC3 cells during the five days of co-culture.

### TUS do not affect the morphology or stemness of transfected MSCs

**Figure 4:**
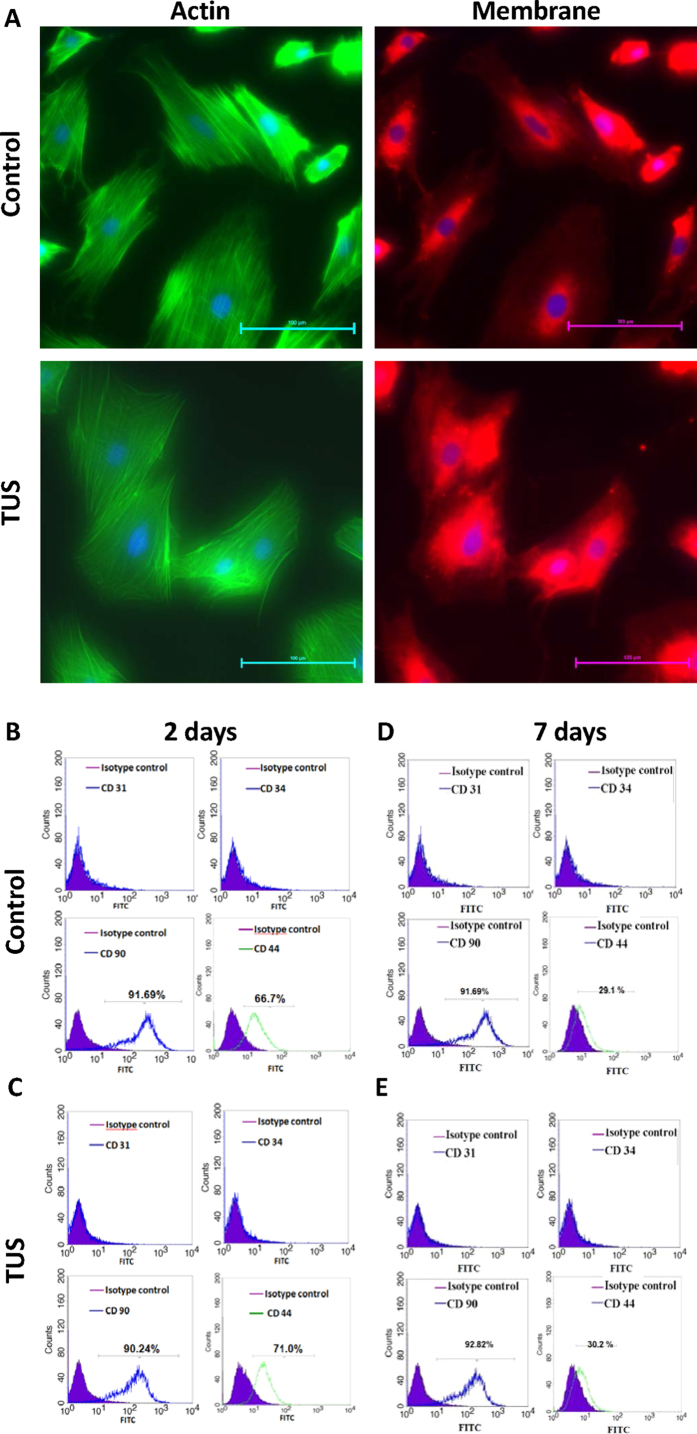
Effect of TUS on the morphology and stemness of the transfected MSCs. (**A**) Effect of TUS + USCA on cell morphology. Representative fluorescent images of actin (green), membrane (red) and nucleus (blue) of TUS treated and untreated MSCs. (**B**–**E**) FACS analyses of MSC cell surface markers (CD31, CD34, CD90 and CD44) 2 (**B**) and 7 (**D**) days post TUS transfection. All markers were compared to their isotype control.

To evaluate whether the energy and bioeffects of TUS lead to a change in cell morphology, MSCs were imaged under a fluorescent microscope following exposure to TUS with USCA and compared to untreated control MSCs (Fig. 4A). Cells were stained with DiI, phalloidin-FITC and Hoechst in order to reveal any effects on cell membrane, actin filaments and nuclei, respectively. Figure 4A demonstrates that no change in the nucleus shape was induced by TUS application. Actin fibers, both in TUS-treated cells and untreated cells were long, fibroblast-like shaped and filled the cell’s entire cytoplasm. Furthermore, no effect of TUS application on cell membrane was detected under higher magnification (Fig. 4A).

To evaluate whether TUS application affects the stemness of MSCs, cells at 2 and 7 days post TUS + USCA application were analyzed for MSC cell surface markers: CD31, CD34, CD90 and CD44, and compared to untreated cells that served as control. Our results show that the treated and untreated cells were positive for CD90 and CD44 and negative for CD31 and CD34 at 2 days post TUS application (Fig. 4B,C), and at 7 days post TUS application (Fig. 4D,E).

### TUS-transfection does not alter MSC biodistribution

**Figure 5:**
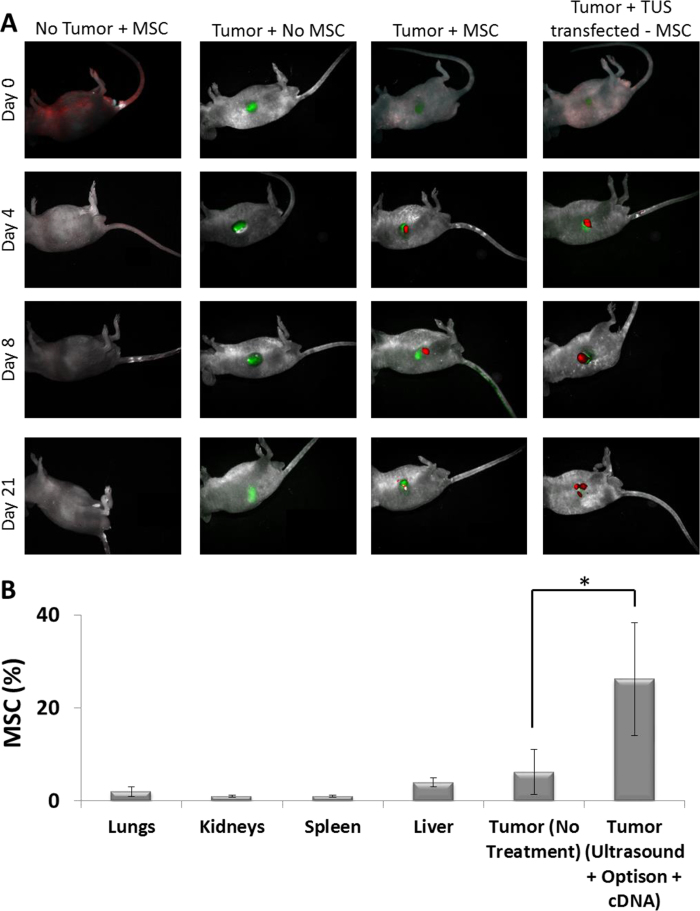
Bio-distribution of TUS-transfected MSCs. (**A**) Representative micrographs of TUS-PEX-transfected MSCs (red) in tumor (green) bearing mice. Cells were administered I.V. and visualized using a whole-body Maestro *in vivo* imaging system^TM^. (**B**) % of MSCs in the tumor and other organs of tumor-bearing mice after I.V. administration of TUS-PEX-transfected MSCs. Cells were analyzed by flow-cytometry using PE anti-CD90 and results are presented as mean ± SD *p < 0.01, n = 5.

To study the biodistribution of TUS-PEX-transfected MSCs, cells were administered I.V. to tumor-bearing mice and visualized using a whole-body Maestro *in vivo* imaging system. The images taken using the green (tumor) and red (MSC) channels clearly showed that immediately after the administration (Day 0, Fig. 5A), the MSCs can be found all over the body, in all the MSC-administered groups (with and without tumor) but not in the untreated control (Tumor + No MSC, Fig. 5A). Four days post administration, red fluorescent MSCs can be found mainly around the tumor, with little traces in other organs. The accumulation of MSCs around the tumor becomes clearer 8 days post administration, when the MSCs can be found only around the tumor site, and no traces are detected in other organs. The MSCs can be found around the tumor site even after 21 days (Fig. 5A).

To quantify these results, an additional experiment was conducted, in which mice were sacrificed 21 days post MSC administration and their tumors and filtrating organs (spleen, kidneys, liver, and lungs) were harvested and analyzed for the presence of MSCs by flow-cytometry. The results revealed a significant accumulation of MSCs in the tumors of mice, which were administered with TUS-treated MSCs (26%) compared to tumors of untreated mice (6%, p < 0.01, Fig. 5B). In addition, in all treatment groups, there was no significant accumulation of MSCs in other organs.

### A single treatment with MSCs that were TUS + USCA-transfected with pPEX leads to tumor inhibition

**Figure 6:**
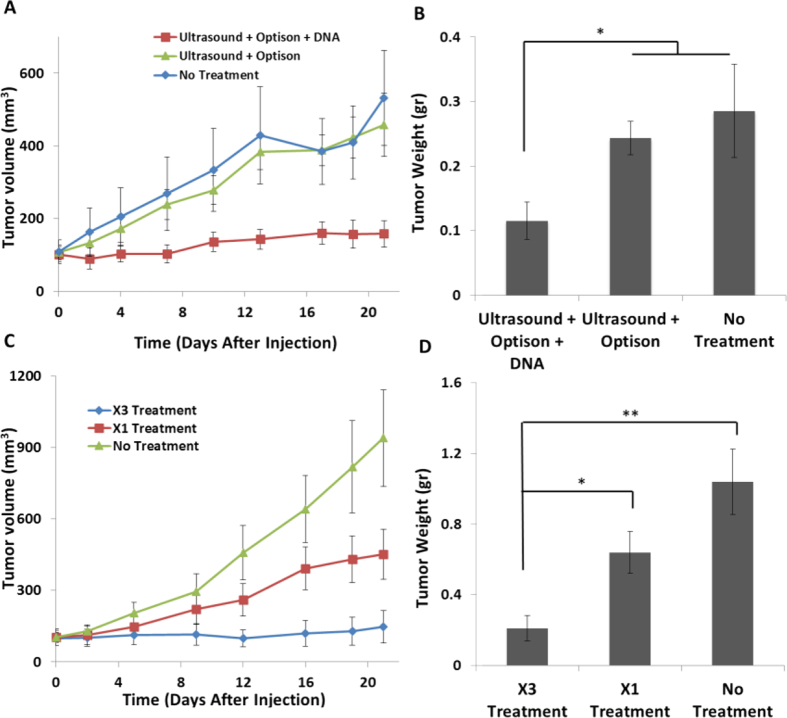
*In vivo* efficacy studies using single (**A**,**B**) and multiple (**C**,**D**) treatments with TUS-transfected MSCs. (**A**,**C**) Average tumor volume at different time points following treatment, and (**B**,**C**) tumor weight 21 days following treatment. Results are presented as mean ± SE *p < 0.05, **p < 0.001, n = 8.

The administration of MSCs that were TUS + USCA-transfected with pPEX led to a significant inhibition in tumor growth 21 days post injection. The result of treatment was an average tumor volume of 158 ± 36 mm^3^ compared to 532 ± 131 mm^3^ in the control group (p < 0.01) and 458 ± 86 mm^3^ in the group administered with MSCs treated with TUS and USCA (i.e., no pDNA-PEX, p < 0.01, Fig. 6A). Tumor *weight* was also significantly lower (0.12 ± 0.03 gr) in mice receiving a single administration of MSCs that were TUS + USCA-transfected with pDNA-PEX compared to TUS + USCA only (0.24 ± 0.03 gr, p < 0.05) or non-treated control cells (0.29 ± 0.07 gr, Fig. 6B).

### Multiple treatments with MSCs that were TUS + USCA-transfected with pPEX enhances tumor inhibition

To assess whether multiple tumor treatments can further enhance the effect obtained by the single dose of MSCs that were TUS + USCA-transfected with pPEX, tumor-bearing mice were injected weekly for three weeks with the same treatment (Fig. 6C). The results clearly demonstrated that tumor growth was significantly inhibited in the multiple treatment group (147 ± 67 mm^3^) compared to the control group (940 ± 203 mm^3^, p < 0.01), as well as the single administration group (451 ± 104 mm^3^, p < 0.05). Tumors *weight* was also significantly lower (Fig. 6D) in the multiple treatment group (0.21 ± 0.07 gr) compared to the tumors of the control group (1.04 ± 0.18 gr, p < 0.001) and the tumors of the single treatment group (0.64 ± 0.11 gr, p < 0.05).

### Histology and IHC of treated mice tumors

**Figure 7:**
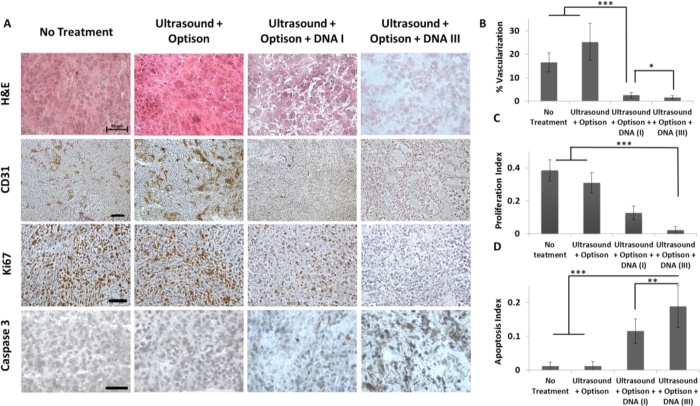
Histology and immunohistochemistry (IHC) of treated tumors. (**A**) Representative micrographs of histology (scale bar: 50 mm) and IHC – vascularization (CD31, scale bar: 100 μm), proliferation (Ki67, scale bar: 100 μm) and apoptosis (caspase 3, scale bar: 50 μm) of tumors from mice after single (DNA I) and multiple treatments (DNA III) compared to control. Quantification of IHC (**B**) % vascularization, (**C**) Proliferation and (**D**) Apoptosis. Results are presented as mean ± SD ***p < 0.001, **p < 0.01, *p < 0.05, n = 10.

IHC of tumors harvested from mice treated with a single administration of MSCs TUS + USCA-transfected with pPEX revealed that the vascularization percentage of treated mice was significantly lower (2.5%) compared to control (16.5%, p < 0.001) and the group administered with MSCs treated with TUS + USCA (25%, p < 0.001). The percent of vascularization in the multiple administration group was even lower than the single administration group (1.5%, p < 0.05, Fig. 7B). The proliferation index was also significantly lower in the multiple administration group (0.02) than in the single administration group (0.13) and the control group (0.39, p < 0.001, Fig. 7C). Apoptosis, on the other hand, was significantly higher in the multiple administration group compared to the single administration group (p < 0.01, Fig. 7D) and compared to control (p < 0.001, Fig. 7D).

## Discussion

MSCs are of much interest in the field of cancer cell-based gene therapy, since MSCs can easily be isolated from patients and cultured *in vitro*, and are also hypo-immunogenic^24^. Most importantly, MSCs are known to target sites of injury, inflammation and tumor after their systemic delivery^25^. Using transfected-MSCs as vehicles of exogenous anti-cancer proteins to the cancer site enables a localized effect with better penetration of the protein at the tumor site. Having a targeted and localized therapeutic effect can significantly reduce the amount of therapeutics needed to inhibit tumor growth, as opposed to systemic protein therapy^26^,^27^. Therefore, MSCs have been utilized as delivery vehicles for anti-cancer therapeutics to treat tumors such as glioma, breast carcinoma, melanoma and lymphoma^28^,^29^,^30^. In most of these studies, however, the MSCs, which are known to resist many of the transfection methods, particularly the non-viral ones^31^, were transduced using different viral-vectors such as lentivirus and adenovirus that are associated with immunogenicity and safety issues^11^,^12^.

In the present study, we demonstrate, for the first time, the use of a TUS approach to transfect MSCs with cDNA expressing an antiangiogenic protein, and establish their efficacy for prospective cancer therapy. We hypothesized that TUS can safely and efficiently transfect MSCs *ex vivo*, preserving the MSCs’ characteristics, and thus allowing their future safe *in vivo* application. TUS is considered a promising non-viral gene delivery method that can be approved for clinical use straightforwardly. The application of TUS enabled the delivery of pDNA not only to cells’ cytoplasm, but more importantly, to the nucleus, resulting in rapid expression of the pDNA^13^,^14^,^15^. As we have previously demonstrated, TUS operates as a mechanical force delivering pDNA to the cell through the cytoplasmatic network and into the nucleus^32^. In the current study, we report on the use of long-term TUS (20 minutes) for the transfection of MSCs, in order to inhibit the growth of prostate cancer, a highly vascularized tumor, which depends on angiogenesis and on MMPs for its growth^33^,^34^. We used a pDNA that encodes for PEX, an inhibitor of angiogenesis. PEX uniqueness lies in its effect on both cancer and endothelial cells as it interacts with α_v_β_3_ integrins and inhibits MMP2^32^,^34^. Several studies demonstrated the *in vivo* efficacy of PEX in inhibiting tumors, mainly glioma^18^,^23^,^35^, and prostate cancer^17^. Using TUS to transfect MSCs with pDNA encoding for PEX can overcome many limitations associated with the delivery of the protein. By transfecting the cells one can localize the pDNA expression at the site targeted by the cells and allow the long-term expression of the inhibitor at the tumor site. Moreover, the use of such an anti-angiogenic gene delivery approach avoids the problems of stability associated with delivery of sensitive proteins. Most importantly, the use of TUS, a technology that does not require the involvement of viral vectors, potentially increases the safety and future clinical applicability of the cells as vehicles, thus further paving the way for their use as a cell-based therapy system^15^,^16^,^36^. Nevertheless, utilizing such technology, characterized by the transmission of mechanical forces as well as energy deposition, raises the questions of whether a non-viral technology can lead to efficient transfection of MSCs without altering the cells, and whether it will be sufficient to allow inhibition of tumor growth.

Our *in vitro* experiments demonstrate that TUS can effectively deliver pDNA encoding for PEX to MSCs, thus enabling its expression and biological activity while preserving high viability levels. This advantage was shown by quantifying the released PEX using ELISA and by the significant inhibition of cell proliferation, migration, and the increase in the number of apoptotic HUVECs, when cultured with conditioned media taken from TUS-transfected MSCs. Moreover, significant inhibition of human prostate cancer cell-line proliferation was achieved after culturing it with conditioned media taken from MSCs that were TUS-transfected with pPEX. We also addressed the effect of Optison^TM^, an USCA, on the transfection level of MSC-pDNA-PEX *in vitro*. Pre-incubation of pDNA-PEX with the USCA prior to TUS-transfection resulted in a significantly higher concentration of PEX in the cell medium and in increased apoptosis of HUVECs. However, when HUVEC and PC3 cells were cultured with conditioned media taken from MSCs that were TUS + USCA-transfected with pPEX, only a non-significant inhibition of cell proliferation was achieved, compared to treatment without USCA. These results were unexpected, since addition of USCA significantly increased both the *in vitro* transfection and the expression of PEX from TUS-transfected cells, as was demonstrated previously by our group and others^20^,^21^,^36^,^37^. Moreover, the effect of PEX was shown to be dose-dependent^17^,^18^; thus, an increase in transfection should result in increased inhibition of HUVEC and PC3 proliferation.

As PEX has a relatively short half-life time, we assumed that some of the PEX in the conditioned media was no longer active when used to treat the cells. To test this hypothesis, we designed a new set of experiments in which the direct effect of the secreted PEX was tested by co-culturing the TUS-transfected MSCs with PC3 cells. In these experiments, the direct inhibiting effect of the secreted PEX from the TUS-transfected MSCs on the proliferation of PC3 cells was significantly higher than when tested with conditioned media. Thus, these results suggest that the secreted PEX has much more potent biological activity than the one shown using conditioned media. However, by co-culturing the TUS-transfected MSCs with PC3 cells, the PEX short half-life was no longer hindering its biological efficacy and the full effect was revealed. Zhang *et al*.^38^, in their work, have also shown that when transduced MSCs (by lentivirus) were co-cultured with PC3 cells, the PC3 growth was inhibited by 50%. Additionally, Li *et al*.^39^ succeeded in transfecting MSCs with ultrasound (0.6 W/cm^2^, 1 MHz, 10%DC, 30 sec) with the addition of USCA and polyethylenimine (PEI) *in vitro*. The transfection was 38-fold higher compared to control and the MSCs maintained their capability of multi-directional differentiation and reproductive activity. These results provide promising data on the ability to transfect MSCs using non-viral methods; however, no *in vivo* studies were reported. To use the transfected-MSCs in future *in vivo* applications, we also studied the long-term effects of the TUS on the transfected-MSCs, confirming that the energy attributed by TUS application does not affect the cell morphology or stemness. Our results clearly demonstrate that TUS application did not affect the nucleus shape and/or the actin fibers’ structure. Further, TUS did not affect the cells’ membrane shape or margins. This is consistent with our previous reports, which demonstrated that TUS bioeffects are reversible and all changes were reversed in a period of 24 hours post TUS^40^.

To assess the biodistribution of the TUS-transfected MSCs and to study whether the application of TUS affect MSCs’ homing capabilities, mice with and without tumors were administered with untreated MSCs and MSCs that were TUS + USCA-transfected with pDNA-PEX, and imaged. From these results, it is clear that immediately after administration, the injected MSCs circulate in the blood stream, as also demonstrated in other studies^41^,^42^,^43^. However, four days after administration, MSCs were mainly located at the tumor site, evidently due to their homing ability, and remained in the tumor bed even 21 days after administration. Moreover, there is no difference in the distribution of TUS-transfected MSCs and non-transfected MSCs. These results, along with the quantification MSCs accumulated in different organs, indicate that TUS does not alter the biodistribution of MSCs or their natural homing ability. Additionally, the fact that the transfected-MSCs can be found in the tumor microenvironment, 21 days post administration, and not in other organs motivates the future clinical use of this application. Based on these results, we performed efficacy studies in which tumor-bearing animals were administered with TUS-pPEX-transfected MSCs. In these studies a single I.V. administration of MSCs that were TUS + USCA-transfected with pPEX was sufficient to dramatically inhibit tumor growth. Similar results were presented by Zhang *et al*.^38^, who transduced MSCs using lentivirus to express TNF-α, and achieved 60% reduction in tumor volume four weeks after treatment of prostate cancer. Our IHC results revealed that in mice administered with TUS-transfected MSCs, the angiogenic index and proliferation rate markedly decreased, whereas apoptosis was significantly higher, indicating a lower grade of PC3 tumor^34^,^44^. Altogether, these results revealed that the TUS-transfected MSCs reached the tumor site and secreted PEX that was biologically active and efficaciously inhibited tumor growth *in vivo*.

Since a single administration of TUS-transfected MSCs showed their accumulation at the tumor site, and since administration of transfected MSCs is a non-invasive, non-immunogenic process and safe in terms of tissue integrity^2^,^3^, multiple administrations of TUS-transfected MSCs should not pose major limitations or side effects, although this is an ongoing debate in the literature^45^,^46^,^47^,^48^,^49^,^50^. Multiple administrations of TUS-transfected MSCs may be clinically relevant when a more prolonged treatment or a more significant inhibition of aggressive tumor growth is needed. In their paper, Altanerova *et al*.^51^ used adipose-tissue-derived MSCs transduced by retrovirus to express the suicide gene cytosine deaminase/uracil phosphoribosyl-transferase. They showed that by repeated administration of the therapeutic MSCs to the brain of glioma-bearing mice, the survival percent increased by 25%. Therefore, we hypothesized that multiple administrations may enable us to increase the duration of protein expression, thus achieving even more effective tumor inhibition. To validate this hypothesis, an additional set of experiments was performed in which mice received multiple administrations (once a week for three weeks) of MSCs that were TUS + USCA-transfected with pPEX. These multiple administrations led to an even stronger tumor inhibition of more than 84%, which was further supported by additional significant reduction in the proliferation and angiogenic indices and increased apoptosis compared to mice singly administered with TUS-transfected MSCs. In our previous study^17^, we showed that repeated treatments of TUS with intratumoral injections of pDNA-PEX and USCA led to an inhibition of 80% in tumor volume. Here, we show a similar level of inhibition with non-invasive I.V. administration, apparently due to the MSCs’ homing ability and their better penetration of tumors.

In conclusion, the present study demonstrates, for the first time, the effectiveness of TUS-transfected MSCs as a combination of cell-based therapy and a non-viral gene-delivery approach for the treatment of cancer *in vivo*. Such an approach allows better localization of the protein expression and its duration while avoiding safety issues associated with viral vectors. Furthermore, based on the MSCs’ homing ability to a variety of cancers^27^,^52^,^53^,^54^,^55^,^56^,^57^,^58^,^59^, this platform can serve as a universal therapeutic delivery vehicle targeting various cancers, and using diverse therapeutic proteins. Most importantly, this approach can facilitate the prospective use of MSCs as a cell-based gene-delivery platform in the clinic.

## Materials and Methods

### Cells and Mediums

Rat bone-marrow derived MSCs (Lonza, Basel, Switzerland) were cultured in Dulbeco’s Modified Eagle’s Medium (DMEM) low-glucose (Sigma-Aldrich) and were used within 7–8 passages. Human prostate cancer cell-line, PC3 (ATCC: #CRL-1435), was cultured in HAM/F-12 nutrient-mixture (Sigma-Aldrich). Human umbilical vein endothelial cells (HUVEC, Lonza) were cultured in EGM-MV Medium (Lonza). Media were supplemented with 10% Gibco FBS, 1% Pen-Strep, and 0.4% Fungizone, all procured from Invitrogen, Carlsbad, CA. Cultures were maintained at 37 °C humidified incubator with 5% CO_2_.

### Plasmid

Expression plasmid DNA (pDNA) for human hemopexine-like domain, pTracer-PEX (pPEX), was constructed by sub-cloning the PEX-cDNA into pTracer plasmid (Invitrogen) using EcoRI and NheI restriction enzymes (Takara-Bio, Japan). The pDNAs were amplified and purified using PureLink HiPure Plasmid Maxiprep Kit (Invitrogen).

### Ultrasound-MSCs transfection

The ultrasound apparatus used for all the experiments is a TUS, which operates at a frequency of 1 MHz and 2 cm^2^ surface area, intensity ranging from 0.1 W/cm^2^ to 2 W/cm^2^ with different duty-cycles (%DC) (UltraMax, XLTEK, Ontario, Canada). The coupling quality and total energy delivered were monitored at all times.

#### Ultrasound setup and *in vitro* preparation of cells for TUS

The transducer was immersed directly into a 6-well plate, plated with 10^5^ cells; the transducer was fixed in one place. The plate was placed on a wet rubber platform to assure full contact between the plate and the rubber. The effect of USCA on transfection was evaluated using Optison^TM^ (Perflutren Protein-Type A Microspheres Injectable suspension, USP, GE Healthcare). The pDNA with or without pre-incubation with Optison at 10% (v/v) was added to the cells, and TUS was applied at 20%DC, 2 W/cm^2^ for 20 minutes.

### Enzyme-linked immunosorbent assay (ELISA)

Three days following TUS application, conditioned media of the transfected cells was collected and PEX was detected in the conditioned media using ELISA with primary antibodies against MMP2 and secondary peroxidase-conjugated antibodies (Abcam, Cambridge, MA). PEX was quantified according to a calibration curve using PEX protein as a standard (bioWORLD, Dublin, OH).

### *In vitro* biological activity of PEX transcribed by MSCs post TUS-transfection

The effect of PEX transcribed by TUS-transfected MSCs on HUVECs’ migration, proliferation, and apoptosis was assessed.

#### Migration

HUVEC stably transduced with pGFP were seeded in a 24-well plate and grown to confluence. A linear scratch was formed in the monolayer of the cells. The stripped area was marked, the wells were washed, and a mixture of 1 ml of EGM-MV medium and 1 ml of conditioned media of MSCs three days post transfection with TUS + pPEX or TUS + USCA + pPEX was added to the wells. GFP-HUVEC treated with conditioned media of MSCs that were incubated with pPEX without TUS application served as control. Fluorescent images were taken immediately, 8, and 15 hours later, using a fluorescent microscope (Nikon Eclipse TE 2000-S, Nikon, Germany). Cells that migrated across the drawn line were counted and the means of all field areas from each group were calculated and presented as a percentage of the control.

#### Apoptosis

HUVEC (20,000 cells/well) were seeded in a 24-well plate. Apoptosis was assessed following treatment with a mixture of 1 ml of EGM-MV medium and 1 ml of conditioned media of MSCs three days post transfection with TUS + pPEX and TUS + USCA + pPEX. Apoptosis was measured using propidium-iodide and Annexin-V using MEBCYTO apoptosis kit (Medical & Biological Ltd., Nagoya, Japan), by flow-cytometry according to the manufacturer’s instructions. As controls, cells were treated with conditioned media of MSCs treated with TUS only, pPEX only, and pPEX + USCA only.

#### Proliferation

Proliferation of HUVEC and PC3 cells was assessed following treatment with a mixture of 1 ml of the cells’ medium and 1 ml of the conditioned media of MSCs three days post transfection with TUS + pPEX and TUS + USCA + pPEX. Proliferation was measured using Alamar Blue cell proliferation assay (AbD Serotec, NC, USA) according to the manufacturer’s protocol.

#### Co-culture viability assay

MSCs (20,000 cells/well) that were TUS-transfected with pPEX + USCA were co-cultured in a 6-well plate with PC3 cells (stably transfected with pGFP, 1:1). Fluorescent images of the co-culture were taken every 24 hr for 5 days and the number of the GFP-PC3 cells was calculated, and compared to control, MSCs treated with pPEX only.

### The effect of TUS on cell morphology

The possible effect of TUS on MSCs’ morphology was evaluated using fluorescent microscopy. MSCs were cultured on cover-slip slides in a 6-well plate (10^5^ cells/well), exposed to TUS + USCA application and then fixed with PFA. Cell membranes were stained with fluorescent membrane tracker DiI and actin filaments were stained with phalloidin-FITC (Sigma-Aldrich). In addition, cell nuclei were stained with Hoechst-33258 (Life Technologies, CA, USA).

### The effect of TUS on MSCs’ stemness

MSCs at different time points post TUS + USCA application were analyzed by flow-cytometry for typical positive and negative MSC surface markers CD90, CD44, CD34, and CD31.

### *In vivo* biodistribution of TUS-transfected MSCs

All animal experiments were performed in accordance with the National Council for Animal Experimentation, Israel Ministry of Health guidelines for the care and use of laboratory animals, and all experimental protocols were approved by the Technion’s Animal Care Committee.

Six weeks-old Athymic nude mice (Harlan Labs, Jerusalem, Israel), were subcutaneously inoculated in the right leg flank with 10^6^ GFP-PC3 cells. Tumor volume was evaluated using the following correlation: Volume[mm^3^] = 0.52*(length[mm]*(width[mm])^2^. Once tumor volume reached an average of 100 mm^3^, the mice were randomized into three groups; 1) Untreated tumor-bearing mice, 2) Tumor-bearing mice administered with MSCs, and 3) Tumor-bearing mice administered with MSCs that were TUS + USCA-transfected with pPEX. An additional group of mice without tumors was administered with MSCs. The mice were injected intravenously (I.V.) with pTomato-MSCs (10^6^ cells/mouse), in 50 μl PBS. *In vivo* imaging using the whole-body Maestro imaging system (Perkin Elmer, Waltham, MA) was performed immediately and 12, 24, 48, 72 and 192 hr post administration of the MSCs.

In a different set of experiments, the mice were sacrificed 21 days post administration and their tumors and filtrating organs (spleen, kidneys, liver, and lungs) were harvested and dissociated into single cell suspensions. Dissociated cells were washed twice and analyzed for MSCs by flow-cytometry using PE anti-CD90 (Biolegend, San Diego, CA).

### *In vivo* efficacy studies using a single treatment of TUS-transfected MSCs

Six-week-old Athymic nude mice (Harlan Labs, Jerusalem, Israel) were subcutaneously inoculated in the flank with 10^6^ PC3 cells. Once tumors volume reached an average of 100 mm^3^, the mice were divided randomly into 3 groups; 1) No treatment, 2) Mice administered with TUS + USCA treated MSCs, and 3) Mice administered with MSCs that were TUS + USCA-transfected with pPEX. Mice were injected I.V. with the MSCs (10^6^ cells/mice) suspended in 50 μl PBS. The mice were sacrificed 21 days post administration and tumor volume and weight were measured.

### *In vivo* efficacy studies using multiple treatments with TUS-transfected MSCs

Based on the single treatment experiments, additional *in vivo* experiments were performed in which the mice were subjected to repeated administration of MSCs that were TUS + USCA-transfected with pPEX. The TUS-transfected MSCs were administered I.V. once a week for three weeks (10^6^ cells per mouse per administration). The experiments included the following groups (8 mice per group): 1) No treatment 2) Single treatment of MSCs that were TUS + USCA-transfected with pPEX, and 3) Repeated treatments of MSCs that were TUS + USCA-transfected with pPEX. Tumor volume and weight were measured and the tumors were taken for histological analyses.

### Histological analysis of harvested tumors

Harvested tumors were embedded in OCT (Tissue-Tek, Sakura Fineteck Inc., Torrance, CA), frozen on dry ice, and sliced into 10-μm-thick sections using Lecia^®^ Cryostat (Menheim, Germany). Tumor sections were subjected to histopathological analysis (H&E) and immunohistochemical (IHC) staining for vascularization (CD31, 1:100; BD Biosciences, San Jose, CA), proliferation (Ki-67, 1:100; Lab Vision, Kalamazoo, MI) and apoptosis (Cleaved caspase-3, 1:100; Cell Signaling, Beverly, MA) using the Vectastain Elite ABC kit (Vector Laboratories, Burlingame, CA). Detection was carried out using 3, 3V-diaminobenzidine chromogen (Sigma-Aldrich). Sections were counterstained with hematoxylin (Sigma-Aldrich), dehydrated in ethanol, and mounted with glass cover-slips. Negative control slides were obtained by omitting the primary antibody. Apoptosis and proliferation were quantified by determining the percentage of positively stained cells (stained cells divided by total nuclei) in 10 randomly chosen fields per tissue section at 200X magnification. Microvessel density was determined by NIS Elements image analysis software (NIKON Instruments, Amstelveen, The Netherlands) using 10 randomly chosen fields per section at 200X magnification.

### Statistics

Results are presented as mean ± SD or mean ± SE as specified for each experiment of at least three replicates. Statistical significance in the differences of the means was evaluated by two-tailed t-test. Analysis of variance (ANOVA) was used to test the significance of differences among groups using SAS^TM^ JMP 6 software (Caty, NC).

## Additional Information

**How to cite this article**: Haber, T. *et al*. Ultrasound-Mediated Mesenchymal Stem Cells Transfection as a Targeted Cancer Therapy Platform. *Sci. Rep.* **7**, 42046; doi: 10.1038/srep42046 (2017).

**Publisher's note:** Springer Nature remains neutral with regard to jurisdictional claims in published maps and institutional affiliations.

## Supplementary Material

Supplementary Figure 1
