# Supplementary material for: Ultrasound-Mediated Mesenchymal Stem Cells Transfection as a Targeted Cancer Therapy Platform
Source: Sci Rep. 2017 Feb 7;7:42046. doi: 10.1038/srep42046 (PMC5294424; doi:10.1038/srep42046)
Supplement: Supplementary Figure 1 [file srep42046-s1.pdf]

Supplementary Materials for

Ultrasound-Mediated Mesenchymal Stem Cells Transfection as a Targeted Cancer Therapy Platform

Tom Haber, Limor Baruch and Marcelle Machluf*

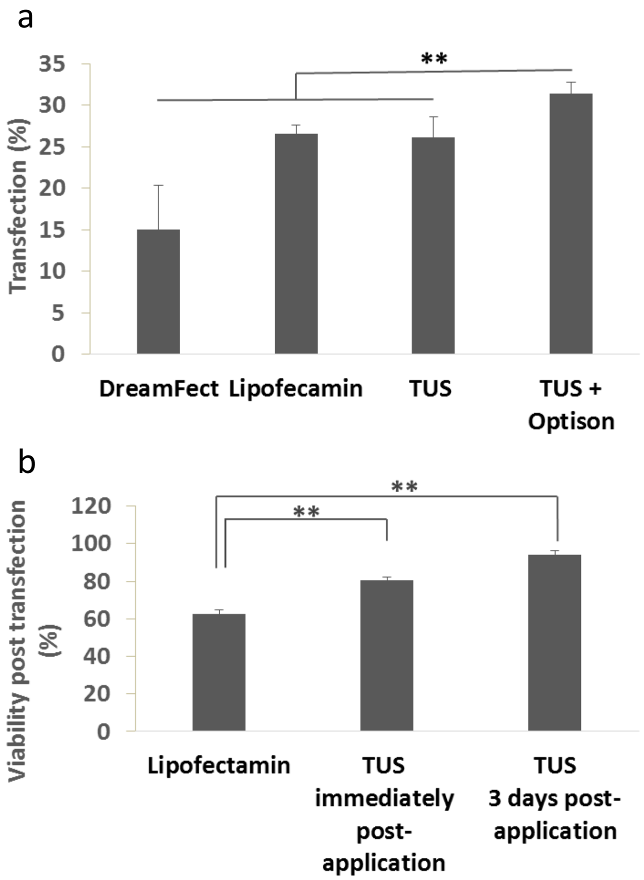

Supplementary Figure 1: MSCs transfection using different methods. (a) The percentage of transfected MSCs following transfection using two popular commercial reagents: Lipofectamine™ (Thermo Fisher Scientific) and DreamFect™ (OZ Biosciences), and TUS with or without USCA. (b) MSC viability post transfection using Lipofectamine™ and TUS. **p<0.01.
